# Mortality and functional outcome after surgical evacuation of traumatic acute subdural hematomas in octa- and nonagenarians

**DOI:** 10.1007/s00068-020-01419-9

**Published:** 2020-06-27

**Authors:** Alexander Younsi, Jessica Fischer, Cleo Habel, Lennart Riemann, Moritz Scherer, Andreas Unterberg, Klaus Zweckberger

**Affiliations:** Department of Neurosurgery, University Hospital Heidelberg, University of Heidelberg, INF 400, 69120 Heidelberg, Germany

**Keywords:** TBI, Acute subdural hematoma, Surgical evacuation, Elderly patients, Octogenarians, Quality of life, Outcome

## Abstract

**Purpose:**

The incidence of acute subdural hematomas (aSDH) is rising. However, beneficial effects of surgery for the oldest aSDH patients remain unclear. We hence describe the postoperative outcome of octa- and nonagenarians with aSDH in comparison to a younger patient cohort.

**Methods:**

Patients aged ≥ 80 years surgically treated for traumatic aSDH at a single institution between 2006 and 2016 were retrospectively reviewed. Clinical and imaging variables were assessed, and univariate analysis was performed to identify factors predicting outcome at discharge. Results were compared to a cohort of younger aSDH patients and statistical analysis was performed. Long-term outcome was prospectively evaluated with the GOSE and QOLIBRI.

**Results:**

27 aSDH patients aged ≥ 80 years were identified. On admission, 41% were in a comatose state and in-hospital mortality was 33%. At discharge, 22% had a favorable outcome (GOS 4 + 5). In univariate statistical analysis, better neurological status (GCS > 8), ≤ 1 comorbidity and smaller aSDH volumes were significant predictors for a favorable outcome. Comparison to 27 younger aSDH patients revealed significant differences in the prevalence of comorbidities and antithrombotics. At long-term follow-up, quality of life of aSDH patients was reduced (median QOLIBRI 54%).

**Conclusion:**

Outcome after surgical treatment of aSDH in octa- and nonagenarians is not detrimental per se. Predictors for a favorable outcome are a non-comatose state on admission (GCS > 8), ≤ 1 preexisting comorbidity and a lower aSDH volume in patients aged ≥ 80 years. In individual patients, surgical evacuation of aSDH might remain a treatment option even in high ages.

## Background

Traumatic brain injuries represent the leading cause of death and disability in patients aged < 45 years, [1, 2] and in this heterogenous field, acute subdural hematoma (aSDH) is considered the most lethal injury [3]. However, demographic studies indicate senescence of the population; while people aged > 65 years accounted for 22% of the total population in 2018, they might represent one third of the total population by 2040 [4]. Accordingly, the age of patients diagnosed with traumatic brain injury (TBI) is increasing [5]. Considering the extended life expectancy with increasing frailty, comorbidities and rate of antithrombotic drug use on the one hand and the growing desire for mobility and autonomy of the older population on the other hand, the incidence of subdural hematomas in the elderly will be a growing problem, encompassing medical, social and financial domains [6].

Nevertheless, treatment decisions for aSDH are mainly based on studies performed on younger populations, disregarding comorbidities and the use of antithrombotic drugs in elderly patients. Moreover, data pertaining neurological outcome in aSDH patients aged ≥ 80 years is still scarce and to date, only two studies have addressed this important topic [7, 8]. This poses new challenges to physicians treating aSDH because well-defined outcome metrics and risk factors for younger aSDH patients do not necessarily apply for this older patient population [7].

Even though improvements in emergency care, neuro-intensive monitoring and surgical techniques have been made in the past decades, recently reported mortality rates for aSDH in the general population still range from 14% in mild traumatic brain injury to 100% in patients with low Glasgow Coma Scale (GCS) scores [9–14], leaving aSDH as one of the unsolved problems in neuro-traumatology.

Moreover, reported outcomes after surgical treatment of aSDH vary considerably with functional recovery being observed in 19–91% of cases even in younger patients [10, 15–17], while data on quality of life after aSDH surgery is generally lacking.

Patient age, pupillary asymmetry and unresponsiveness to light, low GCS scores [18, 19], postoperative seizures, time elapsed until treatment as well as large hematoma volume and midline shift have been described to negatively influence prognosis [15, 16, 20–22]. Unfortunately, most volumetric studies of aSDH date back to the 1990s and lack state-of-the art hematoma segmentation, which weakens these findings [20, 23, 24].

In this light, the decision to perform surgical aSDH evacuation especially in octo- and nonagenarians often remains challenging. With our current study, we, therefore, aimed to describe the clinical course and postoperative outcome of a cohort of the oldest (≥ 80 years), surgically treated aSDH patients in comparison to a younger patient cohort, incorporating state-of-the art segmentation algorithms for volumetric aSDH analysis and including data on long-term quality of life.

## Methods

### Study design

We performed a single center, retrospective study with an additional prospective long-term follow-up analysis. The study was approved by the local ethical committee of our university in conformance to the Declaration of Helsinki and written informed consent for the participation in the prospective outcome analysis was obtained from all included patients [25].

### Participants

The patient data utilized in this study was derived from our institutions electronic hospital records. First, we identified all patients who were diagnosed with aSDH in the period between January 2006 and December 2016. Then, additional inclusion criteria (age ≥ 18 years, aSDH as the main finding on pre-operative imaging, craniotomy or decompressive craniectomy as the surgical procedure for hematoma evacuation) as well as exclusion criteria (spontaneous occurrence of aSDH without a history of trauma, aSDH as a postsurgical complication, acute on chronic SDH, aSDH localized in the posterior fossa, conservative treatment of aSDH or the presence of life limiting comorbidities such as carcinoma) were applied. For further analysis, only the oldest aSDH patients (aged ≥ 80 years) were selected. In an additional step, a group of randomly selected younger aSDH patients (age < 80 years) was formed to compare findings between both age cohorts.

### Data collection

The following clinical parameters were retrospectively reviewed for all patients: age, sex and injury mechanisms, the pupillary status, the clinical symptoms and the GCS on admission. Additionally, details of preexisting comorbidities, usage of antithrombotic drugs and their pre-surgical reversal as well as pre-and postoperative laboratory findings were collected.

For imaging analysis, radiological parameters were assessed on pre-operative computerized tomography (CT) scans. In addition, a cross slice segmentation of volumes was performed on axial CT planes for the supratentorial region using the Medical Imaging Interaction Toolkit (MITK Workbench 2016.11.0, www.Mitk.org), an open source software kit for medical image computation. Segments were traced manually or using a semi-automatic threshold-based region-growing tool. Labeled voxels from every applicable slice were added with the MITK image statistics application to yield volume measures in cubic millimeters.

To assess the surgical procedures and postoperative course, we reviewed the time from admission until surgery, the surgical approach, details of the intensive care unit (ICU) stay as well as intracranial pressure (ICP) monitoring and therapy. If available, radiological findings on postoperative CT scans were additionally assessed. However, postoperative CT scans were only performed when the neurological status of operated patients deteriorated or did not improve.

For outcome assessments, rates of surgical, non-surgical and overall complications as well as the rate of revision surgeries were calculated, and the modality of discharge was noted. Neurological outcome at discharge was evaluated with the GCS and Glasgow Outcome Scale (GOS). Hereby, a poor neurological outcome was defined as GOS 1–3 whereas a favorable outcome was defined as GOS 4–5.

### Prospective outcome measures

For assessment of neurological outcome and quality of life at long-term follow-up, all patients were contacted by mail and the self-rated Quality of Life after Brain Injury questionnaire (QOLIBRI) [26] as well as the Extended Glasgow outcome scale (GOSE) [27] were obtained and supplemented by a telephone interview. We defined a QOLIBRI total score < 65% as an indicator for low or impaired health-related quality of life and GOSE scores of 1–4 as an unfavorable outcome.

Patients who were deceased at the time of the prospective follow-up were excluded from the prospective outcome analysis. Patients who were unwilling or unable to participate in the prospective follow-up or cases in which no information on the current health status could be obtained were defined as lost to follow-up. Written informed consent for the participation in the prospective outcome analysis was obtained from all patients.

### Statistical analysis

Data were analyzed using GraphPad Prism (version 7.0, GraphPad Software Inc.) and are given as median and interquartile range when not stated differently. In addition, univariate analysis was performed using an unpaired t test for comparison of parametric values and Fisher’s exact test for binary variables. To assess the impact of the variables, odds ratios with 95% confidence intervals were calculated. All tests were 2-sided and a *p* value ≤ 0.05 was considered statistically significant.

## Results

### Analysis of the oldest aSDH patients (aged ≥ 80 years)

Between January 2006 and December 2016, 27 patients aged ≥ 80 years with aSDH who were treated at our institution and met the inclusion- and exclusion criteria were identified. Baseline characteristics and outcomes of this particular group of aSDH patients are presented in Table 1.

**Table 1:** Characteristics and outcome of the oldest aSDH patients (≥ 80 years)

	All (no.[%])	Favorable outcome (no.[%])	Unfavorable outcome (no.[%])	OR (95% CI)	*p* value
No. of patients	27	6 (22)	21 (78)		
Demographic factors					
Median age in years (range)	84 (80–93)	84 (80–91)	84 (80–93)		NS
Male sex	16 (60)	3 (50)	13 (62)		NS
Comorbidities					
Arterial hypertension	22 (81.5)	5 (83)	17 (81)		NS
Atrial fibrillation	15 (55.5)	2 (33)	13 (62)		NS
Comorbidities ≥ 5	7 (26)	0 (0)	7 (33)		NS
Comorbidities ≤ 1	5 (18.5)	3 (50)	2 (9.5)	9.5 (1.3–63.3)	0.05^a^
Anticoagulation	23 (85)	4 (67)	19 (90.5)		NS
Clinical presentation and imaging					
Comatose on admission (GCS < 8)	11 (41)	0 (0)	11 (52)	0 (0- 0.7)	0.05^a^
Anisocoria	11 (41)	1 (17)	10 (48)		NS
Midlineshift > 1 cm	12 (44)	1 (17)	11 (52)		NS
Median volume of SDH in ml (IQR)	92 (63–141)	67 (24–91)	118 (71–149)	–	0.05^b^
Rad. Sign of herniation	11 (41)	1 (17)	10 (48)		NS
Reversal of anticoagulation	14 (52)	3 (50)	11 (52)		NS
Details on surgery					
Median time to surgery in min (IQR)	245 (105–1138)	485 (270–488)	208 (97–2036)		NS
Median craniotomy diameter in cm (IQR)	7 (5–8)	7 (5–8)	7 (5–8)		NS
Subdural drains used	15 (55.5)	4 (67)	11 (52)		NS
ICP probe implanted	7 (26)	1 (17)	6 (29)		NS
Median duration of surgery in min (IQR)	125 (101–145)	130 (120–131)	119 (95.5–147)		NS
Postoperative course					
ICU treatment	22 (81.5)	3 (50)	19 (90.5)	0.1 (0.01- 0.8)	0.05^a^
ICP therapy	4 (15)	0 (0)	4 (19)		NS
Surgical complications	7 (26)	0 (0)	7 (33)		NS
Rebleeding	6 (22)	0 (0)	6 (29)		NS
Wound infection	0 (0)	0 (0)	0 (0)		NS
Others	1 (4)	0 (0)	1 (45)		NS
Revision surgery	6 (22)	0 (0)	6 (29)		NS
Non-surgical complications	13 (48)	3 (50)	10 (48)		NS
Remote cerebellar hemorrhage	1 (34)	0 (0)	1 (5)		NS
Cardiovascular	3 (11)	0 (0)	3 (14)		NS
Pneumonia	7 (26)	3 (50)	4 (19)		NS
Respiratory insufficiency	2 (7)	0 (0)	2 (10)		NS
Postoperative seizures	6 (22)	1 (17)	5 (24)		NS
Median hospital stay in days (IQR)	6 (4–10)	8 (5–10)	5 (4–10)		NS
Outcome at discharge					
In-hospital mortality	9 (33)	0(0)	9 (43)		NS
Discharged home	1 (4)	1 (17)	0 (0)		NS
Transferred to other hospital	17 (63)	5 (83)	12 (57)		NS

Favorable outcome = GOS 4 + 5, Unfavorable outcome = GOS 1–3

*GCS *Glasgow coma scale, *SDH* Subdural hematoma, *ICP* Intracranial pressure, *ICU* Intensive care unit, *IQR* Interquartile range, *OR* Odds ratio; *CI* Confidence interval, *NS* not significant

*p* > 0.05

^a^Fisher exact test

^b^Unpaired *t* test

Median age among these 27 oldest aSDH patients was 84 years, ranging from 80 to 93 years, and 59% were male. Preexisting comorbidities were present in 96% of cases, with arterial hypertension being the most common one (81.5%). Seven old aSDH patients (25%) had ≥ 5 comorbidities. Most of the patients (85%) were on antithrombotic therapy when admitted to our hospital. Anticoagulants like Phenprocoumon or antiplatelet agents like Aspirin were used most commonly (39% each), but novel oral anticoagulants (NOACs) were already present in the remaining 18% of cases. Effects of Phenprocoumon were most often reversed with a 4-factor PCC (Prothrombin Complex Concentrate) (in 79% of cases). All patients had suffered from head trauma prior to admission and a comatose status (defined as a GCS score ≤ 8) as well as anisocoria on admission were seen in 41% of patients, respectively.

**Fig. 1:**
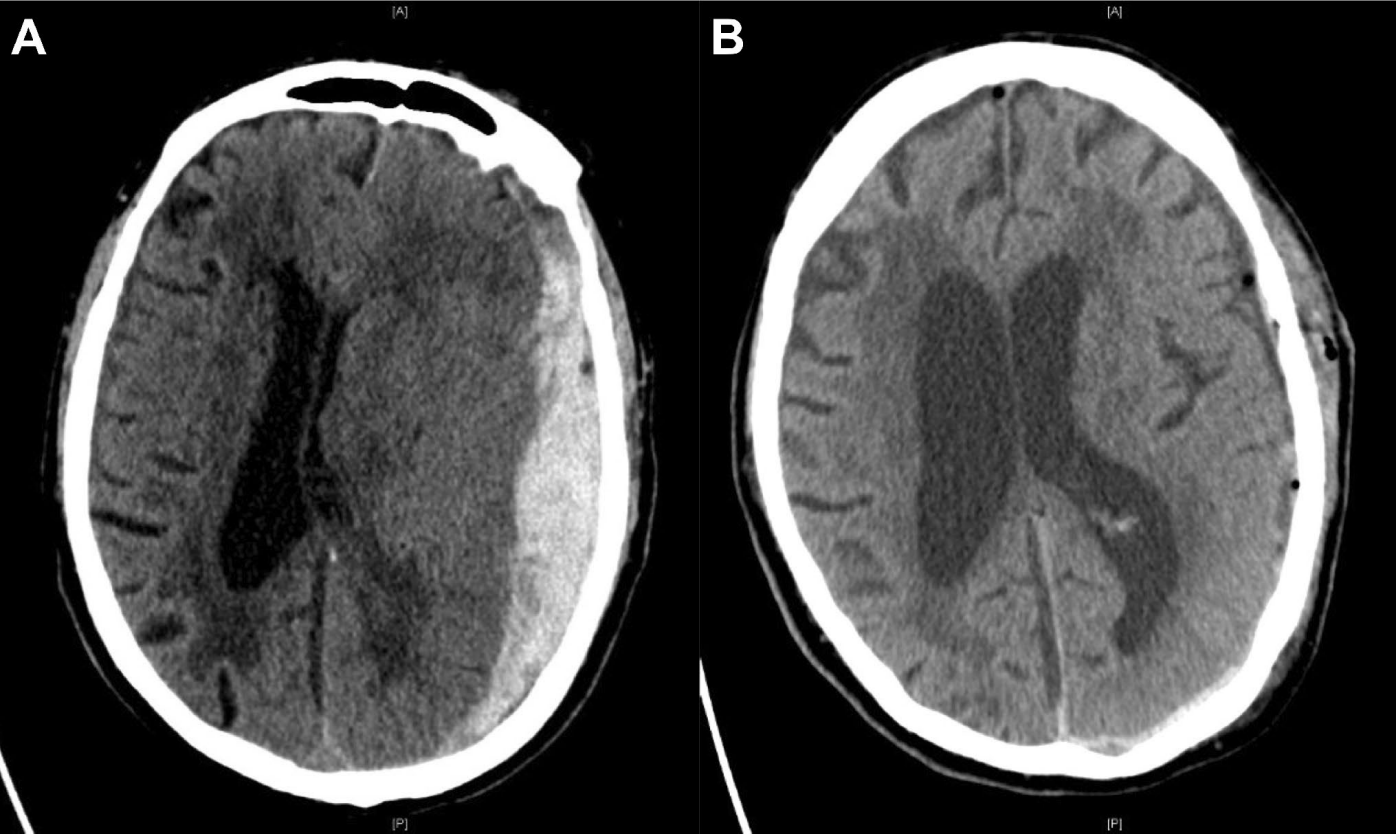
**a** PreOP computed tomography (CT) of an 80-year-old patient showing a left-sided acute subdural hematoma (aSDH) with a max. diameter of 1.9 cm and approximately 1.3 cm midline shift (aSDH volume = 138 ml). **b** PostOP CT showing sufficient evacuation of the left-sided aSDH and declining midline shift. GOS score at discharge was 4

On pre-operative CT scans, left-sided aSDH was present in 44%, right sided in 37% and aSDH on both sides was diagnosed in 19% of cases. A midline shift > 1 cm was detected in almost half of the patients (44%) and median aSDH volume measured by volumetric analysis was 92 ml (63–141 ml). Additional subarachnoid, intracranial and intraventricular hemorrhages were seen in 37%, 41% and 18% of patients, respectively. A representative pre- and postsurgical CT scan is shown in Fig. 1.

Median time elapsed between admission and surgical evacuation of the aSDH was 245 min (105–1138 min). All patients received a craniotomy for surgical evacuation with a median diameter of 7 cm (5–8 cm), measured on postoperative CT scans. Subdural drains were inserted in half of all patients and ICP probes were implanted in 26%. Postsurgically, 82% of patients were treated on the ICU, for a median of 5 days (3–6 days). Additional treatment for elevated ICP was implemented in 15% of cases during ICU treatment. Postoperative CT scans further revealed recurrent bleedings in 6 patients (22%) of which four required revision surgeries. Secondary implantation of an external ventricular drain (EVD) and removal of a stuck subdural drain accounted for two other revision surgeries.

**Fig. 2:**
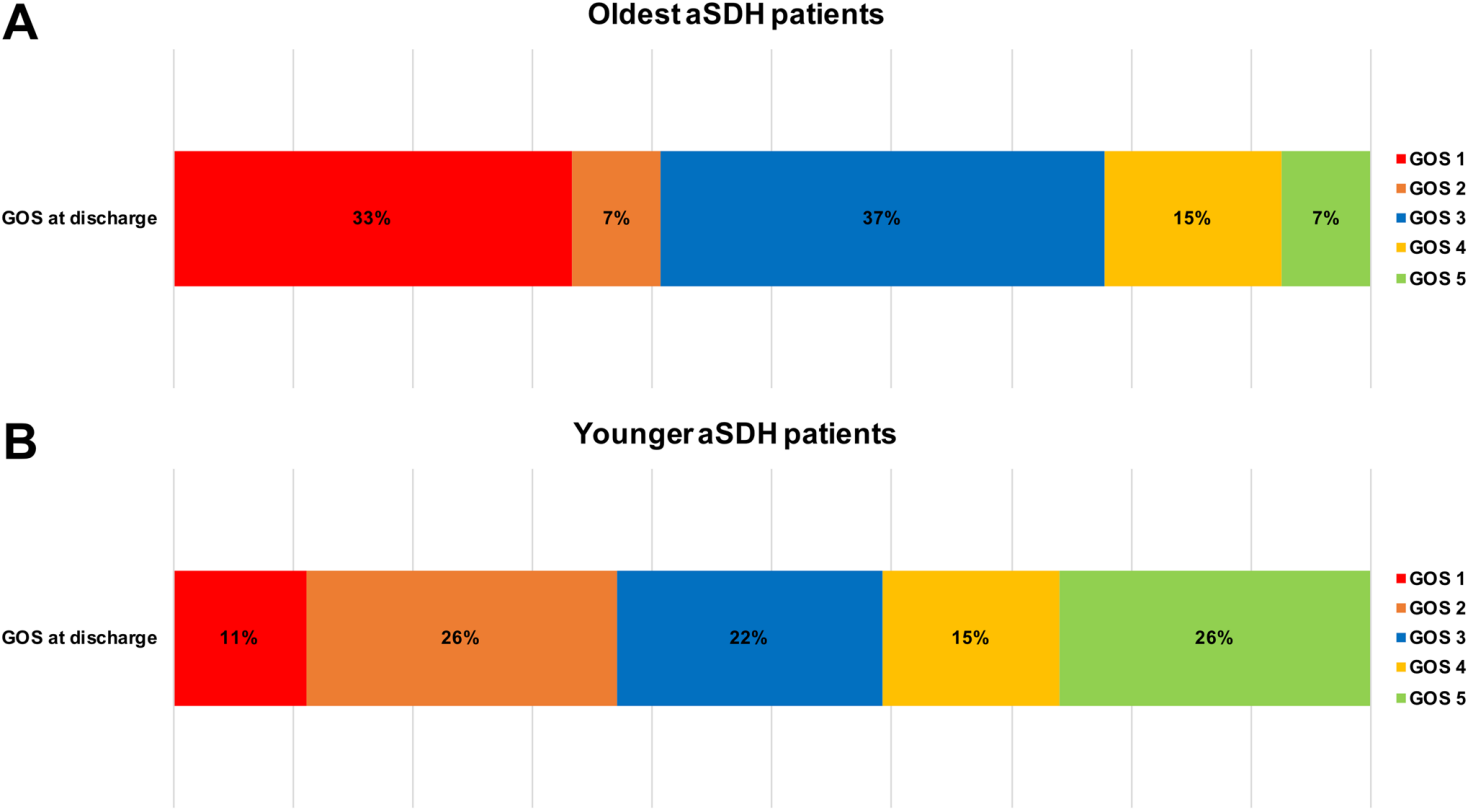
Comparison of the GOS score on discharge for the oldest (age ≧ 80 years; **a**) and younger (**b**) patients, reflecting their functional status

During the hospital stay, pneumonia was the most common non-surgical complication, occurring in one quarter of all patients aged ≥ 80 years, followed by postoperative seizures in 22%. Median duration of hospital stay was 6 (4–10 days). 63% of patients were transferred to another acute care hospital or rehabilitation center, while only one patient was discharged home. One third of the oldest aSDH patients deceased during the in-hospital stay. Only 22% of the patients were discharged with favorable outcomes (GOS 4–5) and 78% had a poor recovery (GOS 1–3). Functional status on discharge, measured by the GOS score is shown in Fig. 2.

In univariate statistical analysis, we found ≤ 1 preexisting comorbidities to be significantly associated with a favorable outcome at discharge (*p* = 0.05). Severity of the injury, defined as a GCS score of 3–8, higher aSDH volume and the necessity of ICU treatment were predictors for a poor functional recovery (*p* = 0.05 each). Surprisingly, the usage of antithrombotic drugs did not significantly influence outcome in the oldest aSDH patients. Neither did anisocoria or radiological signs of herniation correlate with an unfavorable outcome.

### Analysis of the younger aSDH patients

Median age of the 27 randomly selected younger aSDH patients was 65 years, ranging from 19 to 75 years, 59% were male and 78% reported preexisting comorbidities, whereof only 5 patients had ≥ 5 comorbidities. 37% used antithrombotic agents and in 80% of these patients, agents for reversal of the antithrombotic medication were pre-surgically administered.

Almost half of the patients of the younger group were comatose on admission (44%). All patients received CT scans on admission, revealing right and left-sided aSDH with the same frequency. Median maximal diameter of the hemorrhages was 14 mm (11–19 mm) and the calculated median hematoma volume was 71 ml (52–105 ml). In addition, radiological signs of herniation were seen in 33% and skull fractures were present in 44% of cases, while one third presented with additional subarachnoid or intracranial hemorrhages (30 and 33%, respectively). For the younger aSDH patients, median time to surgery after admission was 237 min (118–571 min). Surgical evacuation of the aSDH was performed via a craniotomy in 89% of cases and decompressive craniectomies where performed in the remaining 11%. Subdural drainage systems were used in only 37% of cases and ICP probes were implanted in 26%. After surgery, 70% of patients were treated on the neurosurgical ICU for a median of 6 days (2–11 days). Re-bleedings were seen on postoperative CT scans of four patients; one of them simultaneously developed problems with wound healing. Revision surgery was necessary in all of these patients. One additional case of wound healing disorder occurred but was treated conservatively. Median length of hospital stay for younger aSDH patients was 6 days (4–11 days) during which 37% developed pneumonia and 11% had postoperative seizures. The majority of the younger group was transferred to another health care facility (67%) but 22% of the patients could be discharged home. At discharge, 44% showed functional recovery and 56% had poor outcomes. Mortality in the group of the younger aSDH patients was 11%.

In univariate statistical analysis, younger aSDH patients presenting with ≥ 5 preexisting comorbidities had a significantly worse outcome at discharge (*p* = 0.04). Furthermore, a comatose state on admission (*p* = 0.001) as well as increased midline shift and higher volume of the aSDH (*p* = 0.02 each) were significantly associated with an unfavorable outcome. In contrast to the oldest aSDH patients, anisocoria and radiological signs of brain herniation were able to predict a poor outcome on discharge in the younger group (*p* = 0.008 and *p* = 0.019, respectively). As expected, the necessity of ICU treatment and ICP therapy was also significantly associated with an unfavorable outcome (*p* = 0.008 and *p* = 0.0081, respectively). Interestingly, faster time to surgery did not significantly improve outcome at discharge in the younger group, but patients with smaller craniotomy diameters recovered significantly better (*p* = 0.006).

### Comparison of the oldest and the younger aSDH patients and changes of clinical parameters over time

Details of the comparison analysis of both age groups are presented in Table 2. Patients aged ≥ 80 years had significantly more often arterial hypertension and atrial fibrillation compared to the younger group (*p* = 0.01, respectively). This correlated with a highly significant difference in the frequency of antithrombotic drug usage in the older patients (*p* = 0.0006). Parameters of clinical presentation and CT imaging on admission were comparable between the older and the younger group. Interestingly, time to surgery after admission was significantly shorter in the younger aSDH patients (*p* = 0.0336). Applied surgical methods and postsurgical care, however, did not differ significantly between both groups. Even though postsurgical seizures occurred twice as frequently in patients aged ≥ 80 years then in the younger patients, a significant difference was not reached. More importantly, rate of complications and in-hospital mortality showed no significant difference between both age groups either. Similarly, although 44% of the younger and only 22% of the oldest aSDH patients showed a favorable outcome (GOS 4–5) on discharge, this difference remained insignificant.

**Table 2:** Comparison of the oldest (≥ 80 years) and younger aSDH patients

	All (no.[%])	Oldest patients (no.[%])	Younger patients (no.[%])	OR (95% CI)	*p* value
No. of patients	54	27 (50)	27 (50)		
Demographic factors					
Median age in years (range)	78 (19–93)	84 (81–87)	65 (19–75)		> 0.0001^b^
Male sex	32 (59)	16 (59)	16 (59)		NS
Comorbidities					
Arterial hypertension	34 (63)	22 (81.5)	12 (44)	0.2 (0.1–0.6)	0.01^a^
Atrial fibrillation	20 (37)	15 (55.5)	5 (18.5)	0.2 (0.1–0.6)	0.01^a^
Comorbidities ≥ 5	12 (22)	7 (26)	5 (18.5)		NS
Comorbidities ≤ 1	19 (35)	5 (18.5)	14 (52)	4.7 (1.4–14.8)	0.02^a^
Anticoagulation	33 (61)	23 (85)	10 (37)	0.1 (0.03–0.4)	0.0006^a^
Clinical presentation and imaging					
Comatose on admission (GCS < 8)	23 (43)	11 (41)	12 (44)		NS
Anisocoria	18 (33)	11 (41)	7 (26)		NS
Midlineshift > 1 cm	24 (44)	12 (44)	12 (44)		NS
Median volume of SDH in ml (IQR)	71 (52–105)	92 (63–141)	71 (52–105)		NS
Rad. Sign of herniation	20 (37)	11 (41)	9 (33)		NS
Reversal of anticoagulation	22 (41)	14 (52)	8 (30)		NS
Details on surgery					
Median time to surgery in min (IQR)	245 (111–628)	245(105–1138)	237 (118–571)		0.0336^b^
Median craniotomy diameter in cm (IQR)	7 (5–8)	7 (5–8)	7 (5–8)		NS
Subdural drains used	25 (46)	15 (55.5)	10 (37)		NS
ICP probe implanted	14 (26)	7 (26)	7 (26)		NS
Median duration of surgery in min (IQR)	118 (90–141)	125 (101–145)	115 (83–133)		NS
Postoperative course					
ICU treatment	41 (76)	22 (81.5)	19 (70)		NS
ICP therapy	11 (20)	4 (15)	7 (26)		NS
Surgical complications	13 (24)	7 (26)	6 (22)		NS
Rebleeding	10 (18.5)	6 (22)	4 (15)		NS
Wound infection	2 (4)	0 (0)	2 (7)		NS
Others	1 (2)	1 (4)	0 (0)		NS
Revision surgery	11 (20)	6 (22)	5 (18.5)		NS
Non-surgical complications	26 (48)	13 (48)	13 (48)		NS
Remote cerebellar hemorrhage	1 (2)	1 (4)	0 (0)		NS
Cardiovascular	4 (7)	3 (11)	1 (4)		NS
Pneumonia	17 (31.5)	7 (26)	10 (37)		NS
Respiratory insufficiency	4 (7)	2 (7)	2 (7)		NS
Postoperative seizures	9 (17)	6 (22)	3 (11)		NS
Median hospital stay in days (IQR)	6 (4–10)	6 (4–10)	6 (4–11)		NS
Outcome at discharge					
GOS of 4–5 at discharge	18 (33)	6 (22)	12 (44)		NS
In-hospital mortality	12 (22)	9 (33)	3 (11)		NS
Discharged home	7 (13)	1 (4)	6 (22)		NS
Transferred to other hospital	35 (65)	17 (63)	18 (67)		NS

*GCS *Glasgow coma scale, *GOS* Glasgow outcome scale, *SDH* Subdural hematoma, *ICP* Intracranial pressure, *ICU* Intensive care unit, *IQR* Interquartile range, *OR* Odds ratio; *CI* Confidence interval, *NS* not significant

*p* > 0.05

^a^Fisher exact test

^b^Unpaired *t* test

Comparison of the first half of the study duration (January 2006 to June 2011, 66 months) to the second half (July 2011 to December 2016, 66 months) revealed similar findings for most of the clinical parameters in the aSDH patients overall. Relevant differences between the first and the second half of the study period could be observed concerning patient age (68 (48–74, 5) years vs. 80 (66–85) years, *p* = 0.057), gender (78% male vs. 56% male, *p* = 0.2825) and frequency of the usage of antithrombotic drugs (33% vs. 67%, p = 0.1306). Moreover, differences were present concerning the time to surgery after admission (436 (150–620) min vs. 234 (84–651 min), *p* = 0.7372), and the in-hospital mortality (11% vs. 24%, *p* = 0.6652). However, none of those differences reached statistical significance in univariate analysis. Only the median volume of aSDHs was significantly different during the first half of the study compared to the second half (59 (39–88) vs. 92 (54–139) ml, *p* = 0.0113).

### Prospective follow-up examination for health-related quality of life

Of all aSDH patients in our study, 27 were deceased during the follow-up period and 18 were lost to follow-up. The remaining 9 patients were available for prospective follow-up examination after a median of 30 (21–40) months. Seven of those participants were from the younger age group and two were aged 82 and 83 years, respectively. The two patients aged ≥ 80 years were admitted to the hospital with only mild traumatic injury (GCS score 13–14) but left the hospital in a severely disabled state after aSDH evacuation (GOS 3 both). Nevertheless, they described upper moderate disability (GOSE 6) and upper good recovery (GOSE 8), respectively, at the time of follow-up. Assessment of health-related quality of life by the QOLIBRI questionnaire revealed scores of 40% and 56% for the two patients aged ≥ 80 years, indicating impaired quality of life. Clinical parameters and quality of life scores of all prospectively analyzed patients are presented in Table 3.

**Table 3:** Details on clinical status, neurological outcome and QoL of aSDH patients who participated in the long-term follow-up examination

Age (years)	No. of comorbidities	Anisocoria	General complications	GCS on admission	GOS at discharge	GOS at follow-up	GOSE at follow-up	QOLIRBI at follow-up
37	0	No	Pneumonia	3	2	3	Upper SD (4)	40%
62	3	No	None	14	4	5	Upper GR (8)	58%
65	0	No	Pneumonia	3	3	5	Upper GR (8)	62%
67	4	Yes	Pneumonia	3	2	3	Lower SD (3)	54%
71	4	No	Pneumonia	13	4	5	Lower GR (7)	59%
71	3	No	Pneumonia	13	4	3	Lower SD (3)	40%
72	2	Yes	Resp. Insufficiency	3	2	3	Lower SD (3)	44%
82	4	No	Seizure	14	3	4	Upper MD (6)	40%
83	1	Yes	None	13	3	5	Upper GR (8)	56%

*GCS *Glasgow coma scale, *GOS* Glasgow outcome scale, *GOSE* Glasgow outcome scale extended, *QOLIBRI* Quality of life after brain injury, *GR* Good recovery, *SD* Severe disability, *MD* Moderate disability

## Discussion

### Mortality, functional outcome and quality of life after aSDH surgery in the elderly

Sixty-seven percent of the patients aged 80 years or above surgically treated for traumatic aSDH in our study survived the in-hospital stay and the mortality rate for octa- and nonagenarians thus was only 33%. In contrast to our findings, mortality rates of patients aged 60 or above reported by Jennet et al. in 1976 [16] or by Howard et al. in 1989 [20] were substantially higher, with up to 88% and 74%, respectively. Moreover, the mortality rate of younger aSDH patients (18–40 years) reported by Howard et al. was significantly lower  with 18% compared to the older aSDH patients (≥ 65 years) [20]. Since then, increasing age is considered to be an independent predictor for mortality after aSDH, which has also been confirmed in more recent publications [10–12, 28]. While only 11% of the younger aSDH patients died in direct consequence of the hemorrhage in our study, the difference to the oldest patients did not reach statistical significance. Our results could, therefore, be interpreted as a trend towards decreased early mortality of elderly aSDH patients, similar to recent findings by Won et al. who reported a mortality rate of octogenarians of only 28%, [8] These improvements in early survival of older patients after traumatic aSDH might reflect the advancements of surgical treatments and ICU care in the past decades.

Unfortunately, mortality after aSDH is not restricted to the in-hospital stay but rather continuing during the months after the injury. In a study from the 1990s, mortality during a 6 month follow-up period after aSDH was reported to be as high as 50% [19]. In our study with a long follow-up period, a total of 15 additional aSDH patients had died (62.5%) 30 (21–40) months after the injury. Of those, only four were of the younger age group (27%) and 11 (73%) had been ≥ 80 years old when the aSDH had occurred. Higher mortality rates during follow-up in patients aged ≥ 80 years could be regarded as a direct consequence of the advanced age, often accompanied by an increasing number of comorbidities, a higher incidence of medical complications or a greater usage of antithrombotic agents as seen in our study. In addition, decreasing numbers of intact neurons and greater exposure to repetitive insults over a lifetime [29] could be responsible for impaired repair capacity of brain damage in the elderly, leading to a stepwise increase in probability of poor outcome with increasing age [2, 30].

In previous studies, functional recovery of aSDH patients aged ≥ 65 years has been reported in only 9% of cases, [20] questioning the benefit of surgical evacuation of aSDH in the elderly. Non-age specific favorable outcomes were, however, seen in 19–32%, [10, 15] increasing up to 91% in patients with admission GCS scores of 13–15 [17]. In our current study, we found a favorable outcome at discharge in 22% of the oldest aSDH patients, suggesting improved functional recovery in octo- and nonagenarians compared to older reports in the literature [8, 14]. More importantly, outcome at discharge was not significantly different between the young and the oldest aSDH patients. This might in part be related to the fact, that aSDH patients in our study were mostly treated on our highly specialized neuro-intensive care unit with substantial experience in handling more severe and complex neurosurgical cases.

Although the rate of successful prospective follow-up achieved in our study was low (17%), it revealed exemplary cases of long-term recovery after aSDH surgery in the elderly: two patients aged ≥ 80 years who had both been discharged from the hospital in a severely disabled state (GOS 3) had GCS scores of 13 and 14 and showed good recovery (GOSE 6 and 8) at follow-up. On the other hand, quality of life measured by the QOLIBRI score was low or impaired (< 65%) in all aSDH patients who were available for follow-up in our study, including cases with good functional recovery (GOSE 7 and 8), although a significant correlation between QOLIBRI and GOSE scores for patients with traumatic brain injury has been reported in the literature [26]. It, therefore, seems necessary to evaluate health-related quality of life with corresponding measurements such as the QOLIBRI separately from functional recovery to better understand the overall outcome after surgical treatment in aSDH patients.

### Outcome prediction after aSDH surgery in octo- and nonagenarians

Although comorbidities such as arterial hypertension or atrial fibrillation, correlating with a frequent usage of antithrombotic medication, were significantly more common in the oldest than in the younger aSDH patients, they did not individually predict an unfavorable outcome at discharge in our study. We assume that pre-surgical reversal of antithrombotic medication, interdisciplinary treatment on a specialized ICU and standardized treatment protocols may have reduced the risk for complications and (re)bleedings, and thus might play important roles in the improvement of survival of elderly aSDH patients [8, 22]. However, the presence of fewer comorbidities was associated with a favorable outcome at discharge in patients aged 80 years or above whereas preexistence of ≥ 5 comorbidities was linked to an unfavorable outcome in many patients in both age groups, suggesting that the sum of comorbidities and the resulting general health status are indeed able to influence outcome at discharge after surgical aSDH treatment.

The neurological status after injury, mostly reported as the GCS score at admission, has frequently been associated with functional outcome after aSDH surgery [3, 12]. Correspondingly, we were able to confirm a comatose status at admission, defined as a GCS score of 3–8, as a significant predictor for an unfavorable outcome at discharge in the young, but more importantly also in the oldest aSDH patients in our study. This is in accordance with findings of Won et al., who also confirmed the predictive value of the GCS for functional outcome at discharge in octo-and nonagenarians [8].

Interestingly, anisocoria and radiological signs of herniation were only found to be significant predictors for an unfavorable outcome at discharge in the group of younger aSDH patients in our study. In the elderly patients, less severe brain injury without clinical or radiological signs of herniation might have been sufficient to worsen results of the relatively broad outcome score GOS and might therefore have affected this finding. Nevertheless, pupillary abnormalities should be interpreted as signs of severe injury in all aSDH patients [12, 17, 31]. Similarly, a midline shift > 1 cm was only associated with an unfavorable outcome at discharge in the group of younger aSDH patients, suggesting that effects of brain atrophy and wider subdural spaces in patients aged ≥ 80 years might have allowed for more midline shift without having the same impact on outcome.

### Value of volumetric aSDH analysis in outcome prognostication

The association between aSDH volume and outcome was already analyzed in the 1980s and 1990s, [20, 23, 24] with volumes being manually calculated. Favorable outcomes were seen in patients with mean hematoma volumes of 31 ml [23] (< 100 ml [24]) and poor recovery was associated with mean hematoma volumes of 104 ml [23] (> 100 ml [24]). Additionally, Howard et al. [20] described significant differences in mean volumes between patients aged over 65 years (mean 96.2 ± 11.72 ml) and patients aged 18–40 years (21.6 ± 27.7 ml). Volumetric measurements of aSDH were already conducted in 2009, [31] using computer assisted analysis and volumes < 50 ml were associated with higher rates of functional recovery (50%) in comparison to larger bleedings (> 50 ml, 34% functional recovery).

A correlation between larger hematoma volume and worse outcome was also seen in our patient cohort. Patients aged ≥ 80 years who had an unfavorable outcome showed almost twice the size of aSDH volumes than patients with a favorable outcome. This finding was also applicable for the younger aSDH cohort (*p* = 0.02).

Interestingly, the mean aSDH volume was greater in the elderly patient population compared to the younger patients without yielding statistical significance. Nevertheless, this finding might reflect the higher use of antithrombotic drugs in patients aged 80 years and above, making them prone to more extensive traumatic bleedings.

### Ethical considerations

Our current results underline that while a chance for a good functional outcome exists and while the risk of mortality is reduced, surgical evacuation of traumatic aSDHs in octo- and nonagenarians is still leading to high rates of poor outcome and reduced quality of life – circumstances, under which many older patients might not want to further live their lives. The availability of a patient decree outlining the individual treatment choices and wishes should, therefore, always be inquired and in applicable cases, the decision to perform surgery should only be taken in consideration of the patients written will. In light of frequent preexisting conditions such as care dependency or dementia in the elderly population, the suspected will of the patient has to be respected even when no patient decree is existing or present and thus, her or his relatives should be consulted if possible.

### Limitations

Several limitations of our study should be acknowledged. The analysis was performed at a single institution in a retrospective manner over a long period of time (11 years), limiting external validity and posing the risk of changes in the management of aSDH patients. However, comparison of clinical parameters between the first and the second half of the study period revealed no relevant significant differences over time. Nevertheless, the available patient cohort of aSDH patients aged ≥ 80 years was small and the univariate statistical analysis therefore potentially underpowered. In addition, due to the limited number of eligible aSDH patients overall, mathematical matching of the oldest with the younger patients was not feasible and findings in octo- and nonagenerians were compared with randomly selected younger patients instead. Findings of the comparison between both groups have, therefore, to be interpreted with caution. Due to the small sample size, multivariate analysis was also not feasible, making it impossible to rule out that some variables are dependable in our study. Furthermore, we excluded patients in whom the decision to withhold surgical treatment was taken, hereby potentially biasing our results towards better outcomes. Additionally, an even smaller sample size of patients was available for follow-up analysis which limits the general applicability of our results on health-related quality of life. We also did not differentiate between patients undergoing rehabilitation, biasing outcome at follow-up. Broader prospective studies on long-term outcome of elderly aSDH patients with larger cohorts or national as well as international trauma registries are therefore needed to better understand risks and chances of surgical treatment in this patient subgroup.

## Conclusions

Outcome after surgical treatment of aSDH in octa- and nonagenarians compared to a younger population is not detrimental per se. A lower general health status with more comorbidities as well as a higher usage of antithrombotic drugs represented the major differences between younger and the oldest (age ≥ 80 years) aSDH patients in our study. Moreover, the presence of preexisting comorbidities, a more severe brain injury and a higher volume of the aSDH were associated with a worse outcome in patients aged ≥ 80 years. In comparison to previously published studies, we found lower mortality (33%) and increased functional recovery (22%) rates as well as individual cases of upper moderate disability or upper good recovery in long-term follow-up in the oldest aSDH patients. Our findings suggest, that surgical evacuation of aSDH might be a possible treatment concept for patients even in high ages. Nevertheless, the sum of preexisting comorbidities, poor neurological admission scores and morphologic findings on CT scans should be taken into account and treatment decisions should only be made on an individualized basis, taking into the account the specific wishes of the affected patient.
